# Effects of systemic inflammation due to hepatic ischemia-reperfusion injury upon lean or obese visceral adipose tissue

**DOI:** 10.1590/acb370105

**Published:** 2022-03-14

**Authors:** Ligia Fernanda Ferraz, Cintia Rabelo e Paiva Caria, Raquel de Cássia Santos, Marcelo Lima Ribeiro, Alessandra Gambero

**Affiliations:** 1 Fellow Master degree. Universidade São Francisco – Postgraduate Program in Health Science – Bragança Paulista (SP), Brazil; 2 PhD. Universidade Estadual de Campinas – School of Food Engineering – Department of Food and Nutrition – Campinas (SP), Brazil.; 3 PhD. Universidade São Francisco – Postgraduate Program in Health Science – Bragança Paulista (SP), Brazil.; 4 PhD. Pontifícia Universidade Católica de Campinas – Life Science Center – Campinas (SP), Brazil.

**Keywords:** Endotoxins, Interleukin-6, Reperfusion Injury, Tumor Necrosis Factor-Alpha, Mice

## Abstract

**Purpose:**

To evaluate how the induction of liver damage by ischemia and reperfusion affects the adipose tissue of lean and obese mice.

**Methods:**

Lean and diet-induced obese mice were subjected to liver ischemia (30 min) followed by 6 h of reperfusion. The vascular stromal fraction of visceral adipose tissue was analyzed by cytometry, and gene expression was evaluated by an Array assay and by RT-qPCR. Intestinal permeability was assessed by oral administration of fluorescein isothiocyanate (FITC)-dextran and endotoxemia by serum endotoxin measurements using a limulus amebocyte lysate assay.

**Results:**

It was found that, after liver ischemia and reperfusion, there is an infiltration of neutrophils, monocytes, and lymphocytes, as well as an increase in the gene expression that encode cytokines, chemokines and their receptors in the visceral adipose tissue of lean mice. This inflammatory response was associated with the presence of endotoxemia in lean mice. However, these changes were not observed in the visceral adipose tissue of obese mice.

**Conclusions:**

Liver ischemia and reperfusion induce an acute inflammatory response in adipose tissue of lean mice characterized by an intense chemokine induction and leukocyte infiltration; however, inflammatory alterations are already present at baseline in the obese adipose tissue and liver ischemia and reperfusion do not injure further.

## Introduction

Several liver surgical procedures that include resections or transplants, as well as the occurrence of generalized shock, result in periods of liver ischemia. The consequences of ischemia and subsequent reperfusion can range from transient liver dysfunction to total organ damage associated to a systemic inflammation that can result in multiple organ failure^1^. Liver ischemia-reperfusion injury (IRI) involves several and complex mechanisms, with the participation of different cell types and proinflammatory mediators, also varying in relation to the status of the liver, whether steatotic or nonsteatotic, and the temperature at which it occurs, whether warm or cold ischemia^2^.

It is widely known that adipose tissue is responsible for the production of inflammatory mediators in obesity, collectively called adipokines, which contribute to the establishment of chronic, systemic and low-grade inflammation that occurs in obese patients^3^. In other conditions, such as Crohn’s disease, mesenteric adipose tissue becomes hyperplastic and it creeps around the inflamed segments of the small intestine, releasing proinflammatory mediators^4^. Adipocyte death followed by stem cell activation and tissue remodeling is observed when adipose tissue is exposed to hypoxia, as seen in plastic surgery, for example^5^. Mature adipocytes exposed to hypoxia *in vitro* respond with increased gene expression of vascular endothelial growth factor (VEGF), interleukin (IL)-1, IL-8 and tumor necrosis factor (TNF)-α, but also cytoprotective molecules such as protein heat shock (HSP)-70 and nitric oxide synthase^6^. Most experimental approaches evaluated factors produced by adipose tissue, such as lipids, cortisol and adipokines, or the function of adipose tissue itself or adipose-derived stem cells, for its ability to promote liver regeneration^7^^,^ 8. However, little information is available about the repercussions of systemic inflammation induced by liver IRI upon adipose tissue.

The present study evaluates the effects of systemic inflammatory response triggered by liver IRI on visceral adipose tissue (VAT). By employing lean and diet-induced obese mice, valuable information was added about how lean adipose tissue is affected by liver IRI.

## Methods

### Animals, ethics, and surgical procedure

All experiments were approved by the Ethics Committee of Universidade São Francisco, Bragança Paulista (SP) (Protocol 002.11.2015).

Male Swiss mice were acquired from the Multidisciplinary Center for Biological Research, Universidade Estadual de Campinas (CEMIB/UNICAMP). Mice were maintained in a room with controlled humidity, temperature, and 12-h light-dark cycles in collective cages. Mice were fed ad libitum with standard chow (Presence, SP, Brazil) or high-fat diet prepared as described^9^. Obesity was induced by fed mice with high-fat diet for 12 weeks. Lean mice were age-paired. Six hours prior to experimental procedures, the animals were deprived of food.

Mice were properly anaesthetized by intraperitoneal injection (1:2 v/v ketamine 100 mg·mL^–1^ and xylazine 2%) and maintained warmed (37 °C). Liver was exposed by a small abdominal incision and the hepatic artery and the portal vein were clamped during 30 min. Clamp position induces partial ischemia, along with blood flow interruption to the left lateral and median lobes (70% liver lobe ischemia)^10^^,^ 11. Reflow was observed after clamp removal and the incision was sutured. Reperfusion was maintained for 6 h. Sham-operated were submitted to the same procedure without clamp utilization. Animals were maintained under anesthesia during surgical procedure and resubmitted to anesthesia for euthanasia. Each group was composed of 5 mice. Ten lean mice and 10 obese mice were employed.

### Hepatic and systemic inflammation characterization

Myeloperoxidase (MPO) activity was measured through hepatic biopsies. Briefly, biopsies were homogenized in 0.5% (w/v) hexadecyltrimethylammonium bromide in 50 mmol·L^–1^ potassium phosphate buffer, pH 6.0. Fifty μL of each sample were added to 200 μL of o-dianisidine solution (0.167 mg·mL^–1^ o-dianisidine dihydrochloride, 0.0005% hydrogen peroxide in 50 mmol·L^–1^ phosphate buffer, pH 6.0) and the absorbance change read at 460 nm over 5 min using a microplate reader (Multiscan MS, Labsystems). Serum levels of aspartate aminotransferase (AST) was measured (Bioclin, MG, Brazil) to evaluate liver damage and serum TNF-α and IL-1β was quantified by using enzyme-linked immunosorbent assay kits to evaluate systemic inflammation (Raybiotech, GA, USA).

### Stromal vascular fraction from visceral adipose tissue characterization

Epididymal adipose tissue samples were digested in collagenase solution (1 mg·mL^–1^) in PBS for 45 min at 37 °C with constant shaking (100 cycles·min^–1^). Cells were filtered through 180 and 37 μmol·L^–1^ nylon mesh to isolate the stromal vascular fraction (SVF). Cells (10^6^ cells) were incubated for 30 min in the dark at 4 °C with anti-CD45PerCP (leukocytes), anti-CD14FITC/anti-F4/80PE/anti-CD11bPerCP (macrophages), anti-CD11bPerCP/anti-Ly-6CFITC/anti-Ly-6GPE (neutrophils, monocytes), anti-CD19FITC/anti-CD3PE/anti-CD4PerCP (lymphocytes). Isotype-matched murine FITC, PE and PerCP conjugated immunoglobulin were used as controls. Anti-F4/80 was acquired from Affimetrix eBiosciences, CA, USA. Other antibodies were from BD Biosciences Pharmingen, CA, USA. For each sample, 10,000 events were collected on a Guava Easy-Cyte HT (Millipore, CA) cytometer, defining forward scatter, side scatter in a linear scale and FL1, FL2, and FL3 on a logarithmic scale. Five independent samples were analyzed. Light scatter profiles are obtained for each candidate population using InCyte software (Millipore).

### Inflammatory cytokines and receptors PCR Array and RT-qPCR analysis

Epididymal adipose tissue biopsies were submitted to total RNA extraction using TRIzol (Life Technologies, CA, USA). RNA samples were used for cDNA synthesis using the High-Capacity cDNA Archive Kit (Applied Biosystems, CA, USA). This material was used to analyze the expression of 84 genes that encode chemokines and their receptors by the PCR array technique (Inflammatory Cytokines and Receptors RT2 Profiler PCR Array, Qiagen, CA, USA). Using SYBR Green PCR Master Mix (Applied Biosystems), the samples were amplified on the 7300 Real-Time PCR System and analyzed by Qiagen web portal.

For RT-qPCR, samples were amplified and analyzed using the RQ Study Software (Applied Biosystems). The experiments were done in duplicate of four different samples. Primers sequences and gene evaluated in PCR array (Table 1).

**Table 1:** Primer sequences used in the study.

**Primer (mouse)**		**Sequences**
Actb	forward primer reverse primer	*ACGAGGCCCAGAGCAAGAG* *GGTGTGGTGCCAGATCTT*
Il6	forward primer reverse primer	*TCCACGATTTCCCAGAGAAC* *CCGGAGAGGAGACCTCACAG*
Il1b	forward primer reverse primer	*GTACCAGTTGGGGAACTCCTGC* *GAAGATGGGAAAAGCGGTTTG*
Tnf	forward primer reverse primer	*TAGCCAGGAGGGAGAACAGA* *TTTTCTGGAGGGAGATGTGG*
Lep	forward primer reverse primer	*ACCAAACCAAGCATTTTTGC* *CTATGCCACCTTGGTCACCT*
Adipoq	forward primer reverse primer	*GATCTGTGAACTCTGATCCAGTAAG* *AATAAGGGTCAAGGCCTGGAAACAC*

### Intestinal permeability and endotoxemia evaluation

The mice were submitted to liver IRI and after 3 h received orally 4-kDa FITC-dextran (Sigma-Aldrich, MO, USA) 500 mg/kg. After 3 h of FITC-dextran administration, mice were anaesthetized and blood was collected by cardiac puncture. Serum was obtained by centrifugation and analyzed using a fluorimeter (ex. 485 nm and em. 535 nm; Promega Glomax, WI, USA). FITC-dextran concentrations were calculated from a standard curve of FITC-dextran.

Serum endotoxin measurements were performed using a limulus amebocytes lysate (LAL) assay (Pierce LAL Chromogenic Endotoxin Quantitation Kit, Pierce Biotechnology, IL, USA).

### Statistical analysis

Data are expressed as mean ± SEM. Comparisons among groups of data were made using an unpaired Student’s t-test. An associated probability (p-value) of 5% was considered significant.

## Results and discussion

The consequences of liver ischemia and reperfusion have always been considered important elements in the morbidity and mortality resulting from situations of hemorrhagic shock, trauma, resections, and liver transplants. High morbidity and mortality occur because liver ischemia and reperfusion modify the function of many remote organs, such as lung, kidney, intestine, pancreas, adrenals and heart due metabolic and oxidative changes, and inflammatory responses triggered after reperfusion^12^. Using lean and obese mice, a well-described model of partial liver ischemia was performed, followed by 6 h of reperfusion and the inflammatory response on visceral adipose tissue was evaluated.

**Figure 1:**
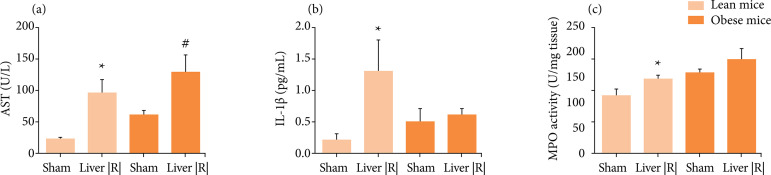
Hepatic and systemic effects in response to 30 min of hepatic ischemia followed by 6 h of hepatic reperfusion in lean and obese mice. Aspartate aminotransferase (AST) serum levels **(a)**, interleukin-1β serum levels **(b)** and myeloperoxidase (MPO) activity in liver samples of lean and obese mice after liver IRI or sham procedure (n = 4–5). *p < 0.05 liver IRI *vs.* sham group.

The data set revealed that AST and IL-1β serum levels, as well liver MPO activity were higher in lean mice submitted to liver IRI when compared with lean mice sham-operated. However, obese mice did not present increase in liver MPO activity and IL-1β serum levels after liver IRI. Furthermore, increased AST serum level was observed after liver IRI procedure (Fig. 1). Obesity *per se* increase AST serum level as well hepatic MPO activity when compared with lean mice (p ≤ 0.01). The results agree with previous literature data from ob/ob mice. Subjects were submitted to 20–40 min of ischemia and 6 h of reperfusion and presented less or similar neutrophil count and a reduced inflammatory response associated to a reduced blood flow in steatotic liver despite the increase of alanine aminotransferase levels when compared with the lean littermates. In the genetic obesity model, Hasegawa *et al*.^13^ suggested that ischemic necrosis is the main mechanism of reperfusion injury in mice steatotic liver.

**Figure 2:**
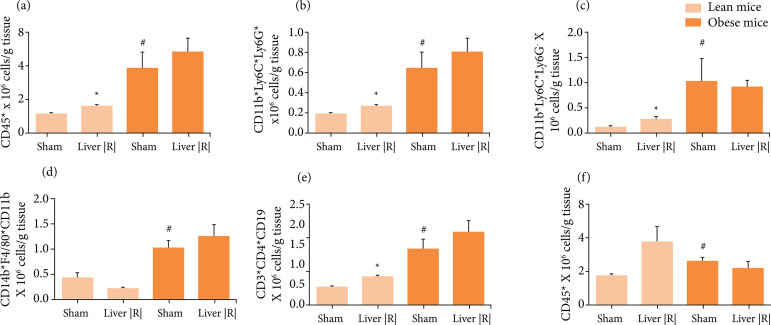
Cytometry analysis of cellular infiltration observed in the stromal vascular fraction of visceral adipose tissue from lean and obese mice after liver IRI or sham procedure (n = 4–5). **(a)** Total leukocytes; **(b)** Neutrophils; **(c)** Monocytes; **(d)** Macrophages; **(e)** T-lymphocytes; f. B-lymphocytes. *p < 0.05 liver IRI *vs.* sham group.

Analysis of SVF from lean VAT revealed that liver IRI induce a significant increase in leukocytes, including neutrophils, monocytes and lymphocytes, but not macrophages, characterizing an acute response. In a long-time protocol of intermittent hypoxia, mimicking sleep apnea condition, leukocyte infiltration in VAT was also observed^14^. It was reported that obesity was able to induce an increase of all leukocytes in SVF of VAT^15^. However, liver IRI was not able to induce additional increases in SVF leukocytes in obese VAT (Fig. 2).

Expression analysis of 84 genes that encode mouse chemokines and their receptors revealed that liver IRI induce an overexpression of 84.5% of genes in lean VAT (Table 2). Considering an intense infiltration of inflammatory cells in adipose tissue of lean mice was observed in response to liver IRI, the increase in the expression of chemokines and their receptors was expected. Accordingly, not only trafficking but the function of neutrophils is coordinated by chemokines during inflammatory conditions^16^. Neutrophils from mice express CXCL1, CXCL2, and CXCL5, but not CXCL8 as human neutrophils^17^. Mouse CXCR1 and CXCR2 are involved in neutrophil recruitment^18^ and CCR1 along with other CC receptors (CCR2, CCR3, CCR5) were also implicated in neutrophil recruitment in different murine disease models^19^^-^ 23. Therefore, it is rational to assume that the increased expression of *Cxcl1* and *Cxcl2*, as well *Ccr1*, *Ccr3*, *Ccr5*, *Cxcr1* and *Cxcr2* observed in adipose tissue may be related to the neutrophils-driven to the inflammatory site. Monocyte chemoattractant protein-1 (MCP-1/*Ccl2*) and additional *Ccl7* and *Ccl8* chemokines are upregulated in adipose tissue after liver IRI. Acting through CCR2, these chemokines recruit monocyte Ly-6C^hi^ during inflammation^24^^,^ 25. T cells are recruited to the liver after ischemia, and hepatic increased expression of *Ccl2, Ccl3, Ccl4, Ccl5, Cxcl2* and *Cxcl10* was observed^26^.

**Table 2:** The mouse inflammatory cytokines and receptors RT2 profiler PCR array from lean liver IRI mice vs. lean sham-operated mice.

**Symbol**	**Description**	Log2*
*Ackr1*	Duffy blood group, chemokine receptor	2.83
*Ackr2*	Chemokine binding protein 2	5.47
*Ackr3*	Chemokine (C-X-C motif) receptor 7	3.24
*Ackr4*	Chemokine (C-C motif) receptor-like 1	2.81
*C5ar1*	Complement component 5a receptor 1	5.01
*Ccl1*	Chemokine (C-C motif) ligand 1	5.01
*Ccl11*	Chemokine (C-C motif) ligand 11	0.21
*Ccl12*	Chemokine (C-C motif) ligand 12	1.68
*Ccl17*	Chemokine (C-C motif) ligand 17	7.33
*Ccl19*	Chemokine (C-C motif) ligand 19	6.99
*Ccl2*	Chemokine (C-C motif) ligand 2	5.02
*Ccl20*	Chemokine (C-C motif) ligand 20	6.42
*Ccl22*	Chemokine (C-C motif) ligand 22	6.83
*Ccl24*	Chemokine (C-C motif) ligand 24	7.13
*Ccl25*	Chemokine (C-C motif) ligand 25	2.88
*Ccl26*	Chemokine (C-C motif) ligand 26	6.50
*Ccl28*	Chemokine (C-C motif) ligand 28	5.84
*Ccl3*	Chemokine (C-C motif) ligand 3	3.87
*Ccl4*	Chemokine (C-C motif) ligand 4	4.29
*Ccl5*	Chemokine (C-C motif) ligand 5	3.37
*Ccl6*	Chemokine (C-C motif) ligand 6	-1.87
*Ccl7*	Chemokine (C-C motif) ligand 7	5.44
*Ccl8*	Chemokine (C-C motif) ligand 8	3.33
*Ccl9*	Chemokine (C-C motif) ligand 9	2.40
*Ccr1*	Chemokine (C-C motif) receptor 1	4.23
*Ccr10*	Chemokine (C-C motif) receptor 10	4.85
*Ccr1l1*	Chemokine (C-C motif) receptor 1-like 1	4.66
*Ccr2*	Chemokine (C-C motif) receptor 2	1.82
*Ccr3*	Chemokine (C-C motif) receptor 3	5.21
*Ccr4*	Chemokine (C-C motif) receptor 4	6.74
*Ccr5*	Chemokine (C-C motif) receptor 5	3.51
*Ccr6*	Chemokine (C-C motif) receptor 6	5.12
*Ccr7*	Chemokine (C-C motif) receptor 7	5.78
*Ccr8*	Chemokine (C-C motif) receptor 8	5.93
*Ccr9*	Chemokine (C-C motif) receptor 9	4.72
*Ccrl2*	Chemokine (C-C motif) receptor-like 2	4.67
*Cmklr1*	Chemokine-like receptor 1	3.26
*Cmtm2a*	CKLF-like MARVEL transmembrane domain containing 2A	5.07
*Cmtm3*	CKLF-like MARVEL transmembrane domain containing 3	2.11
*Cmtm4*	CKLF-like MARVEL transmembrane domain containing 4	0.62
*Cmtm5*	CKLF-like MARVEL transmembrane domain containing 5	6.16
*Cmtm6*	CKLF-like MARVEL transmembrane domain containing 6	0.84
*Cx3cl1*	Chemokine (C-X3-C motif) ligand 1	3.87
*Cx3cr1*	Chemokine (C-X3-C) receptor 1	5.13
*Cxcl1*	Chemokine (C-X-C motif) ligand 1	4.02
*Cxcl10*	Chemokine (C-X-C motif) ligand 10	4.09
*Cxcl11*	Chemokine (C-X-C motif) ligand 11	2.76
*Cxcl12*	Chemokine (C-X-C motif) ligand 12	-0.23
*Cxcl13*	Chemokine (C-X-C motif) ligand 13	5.99
*Cxcl14*	Chemokine (C-X-C motif) ligand 14	2.41
*Cxcl15*	Chemokine (C-X-C motif) ligand 15	6.29
*Cxcl16*	Chemokine (C-X-C motif) ligand 16	3.13
*Cxcl2*	Chemokine (C-X-C motif) ligand 2	4.01
*Cxcl3*	Chemokine (C-X-C motif) ligand 3	6.41
*Cxcl5*	Chemokine (C-X-C motif) ligand 5	1.26
*Cxcl9*	Chemokine (C-X-C motif) ligand 9	4.10
*Cxcr1*	Chemokine (C-X-C motif) receptor 1	4.98
*Cxcr2*	Chemokine (C-X-C motif) receptor 2	6.10
*Cxcr3*	Chemokine (C-X-C motif) receptor 3	5.42
*Cxcr4*	Chemokine (C-X-C motif) receptor 4	3.48
*Cxcr5*	Chemokine (C-X-C motif) receptor 5	5.41
*Cxcr6*	Chemokine (C-X-C motif) receptor 6	4.37
*Fpr1*	Formyl peptide receptor 1	4.74
*Gpr17*	G protein-coupled receptor 17	4.08
*Hif1a*	Hypoxia inducible factor 1, alpha subunit	1.08
*Ifng*	Interferon gamma	5.15
*Il16*	Interleukin 16	3.70
*Il1b*	Interleukin 1 beta	4.06
*Il4*	Interleukin 4	5.29
*Il6*	Interleukin 6	5.93
*Itgam*	Integrin alpha M	1.69
*Itgb2*	Integrin beta 2	1.67
*Mapk1*	Mitogen-activated protein kinase 1	0.21
*Mapk14*	Mitogen-activated protein kinase 14	3.17
*Pf4*	Platelet factor 4	3.60
*Ppbp*	Pro-platelet basic protein	7.33
*Slit2*	Slit homolog 2 (Drosophila)	3.35
*Tgfb1*	Transforming growth factor, beta 1	1.01
*Tlr2*	Toll-like receptor 2	4.60
*Tlr4*	Toll-like receptor 4	3.29
*Tnf*	Tumor necrosis factor	9.10
*Tymp*	Thymidine phosphorylase	4.61
*Xcl1*	Chemokine (C motif) ligand 1	4.98
*Xcr1*	Chemokine (C motif) receptor 1	4.27

The analysis of this work also indicated all these chemokines genes increased in adipose tissue after liver IRI. The receptor CCR7, a ligand for the lymphoid chemokine CCL21 and CCL19 may be an important inducer of lymphocyte migration to atherosclerotic lesions^27^, whereas CCL25, CCL27 and CCL28 are pointed as important chemokines expressed by epithelial cell at specific sites, as small intestine, skin or mucosal, respectively^28^. Indeed, the obtained data revealed a strong overexpression of *Ccr7, Ccl19* and *Ccl28*. Lastly, results also showed that several endothelial cells secreted chemokines genes^29^ were upregulated (*Cxcl1, Cxcl2, Cxcl10, Cxcl9, Ccl2, Ccl3, Ccl5, Ccl7* and *Cx3cl1*), suggesting the participation of vascular cells in the leukocyte recruitment to adipose tissue during liver IRI.

**Figure 3:**
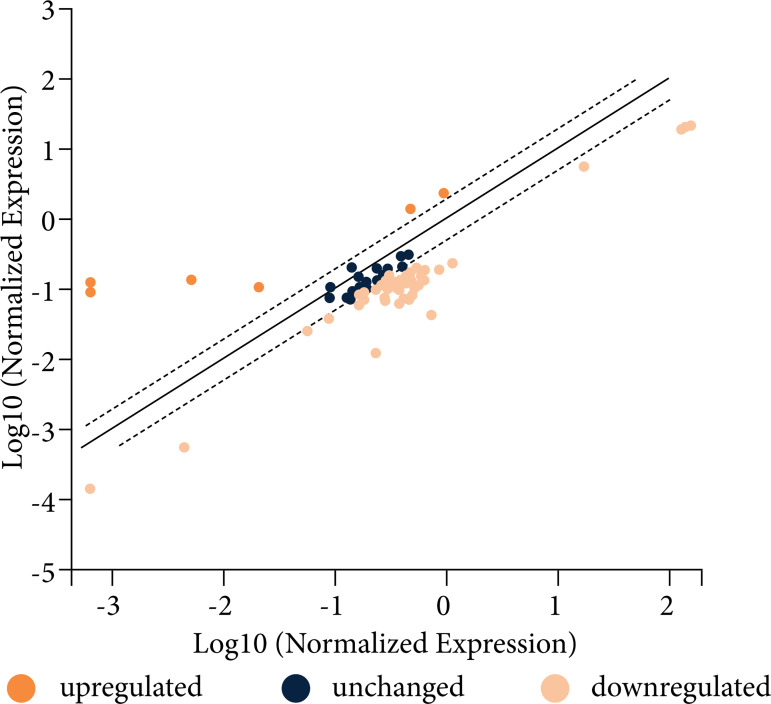
Illustrative scatter plot demonstrating gene expression of VAT from obese mice submitted to liver IRI normalized by gene expression of VAT from obese sham-operated mice. The central line indicates unchanged gene expression. The dotted lines indicate the selected fold regulation threshold. Data points beyond the dotted lines in the upper left and lower right sections meet the selected fold regulation threshold.

A preliminary PCR array analysis revealed that liver IRI did not induce similar response in obese VAT (Fig. 3), as only 4.8% of analyzed genes were upregulated (*AcKr1, Ccr3, Mapk14 and Ppbp*). The induction of liver IRI in obese mice was not able to promote an inflammatory response with the production of mediators that reach the adipose tissue and, therefore, an additional inflammatory response in the obese adipose tissue was not observed. For this reason, additional assessments are for lean animals only. However, it is important to note that several genes encoding chemokines and its receptors that were overexpressed in adipose tissue as a response to liver IRI were reported in previous studies as overexpressed in response to high-fat diet in obese KKAy mice, as *Ccl19, Ccl21, Ccl25, Cxcl2, Cxcl10* and *Ccr7* 30 or in polygenic fat mice, as *Ccl2, Ccl3, Ccl4* and *Ccl7* 31. Our preliminary array data revealed that adipose tissue from obese mice overexpressed *Ccl3, Ccl11, Ccl17, Ccl19, Ccl22, Cxcl9, Cxcl10, Cxcl11, Ccr4, Ccr7* and *Ccr8,* many of which were overexpressed in adipose tissue of lean mice in response to liver IRI.

Fold-Change Log2. Fold-change (2^ (- Delta Delta CT)) is the normalized gene expression (2^ (- Delta CT)) in the liver IRI mice sample divided the normalized gene expression (2^ (- Delta CT)) in the Sham-operated mice sample. Log 2 Fold-change values greater than 2 indicates an upregulation and fold-change values less than –2 indicate down-regulation (n = 2).

**Figure 4:**
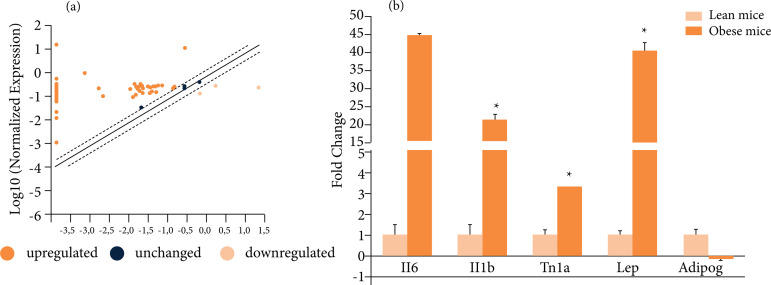
Alterations in the expression of genes encoding cytokines, chemokines and their receptors in adipose tissue **(a)** Illustrative scatter plot demonstrating the gene expression listed on Table 2 of VAT from lean mice submitted to liver IRI normalized by gene expression of VAT from sham-operated mice. The central line indicates unchanged gene expression. The dotted lines indicate the selected fold regulation threshold. Data points beyond the dotted lines in the upper left and lower right sections meet the selected fold regulation threshold. **(b)** Il6, Il1b, Tnf, Lep and adipoq mRNA expression validated by RT-qPCR in VAT of sham and liver IRI mice (n = 4). *p < 0.05 liver IRI *vs.* sham group.

In view of the impossibility of validating all the genes that were upregulated in the array analysis of lean mice by RT-PCR analysis, *Il1b*, *Il6* and *Tnf* gene were chosen because their involvement in sterile inflammation responses^32^^-^ 35. The overexpression of *Il6*, *Il1b* and *Tnf* was confirmed by RT-qPCR, as well as a significant overexpression of *Lep* and no modification of *Adipoq* gene expression in lean VAT due hepatic IRI (Fig. 4), two important adipokines that were not included in the array.

**Figure 5:**
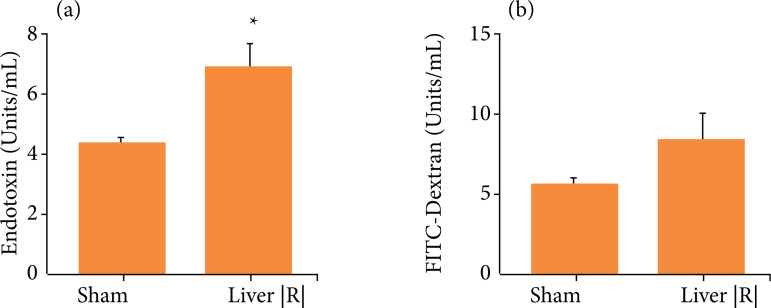
Observed alterations in intestinal permeability after liver IRI or sham procedures in lean mice. **(a)** Endotoxin serum levels; **(b)** FITC-dextran serum levels; (n = 4–5). *p < 0.05 liver IRI *vs.* sham group.

Endotoxin serum levels were increased but serum levels of FITC-dextran were not significantly modified in lean mice after liver IRI (p = 0.14; Fig. 5). Ex vivo exposure of subcutaneous and VAT from lean volunteers to different concentrations of lipopolysaccharide (LPS) was able to produce an inflammatory response with increased expression of Il-6 and TNF-α^36^ and experimental endotoxemia in lean volunteers was also able to induce an inflammatory response with expression of IL-6, TNF-α and chemokines, mainly involved in monocyte recruitment^37^. Accordingly, endotoxemia promoted by liver IRI could be one of the inflammatory stimuli in lean VAT, as described to occur during obesity^38^.

## Conclusion

Lean VAT could be provoked by liver IRI triggering an acute inflammatory response characterized by an intense chemokine induction and leukocyte infiltration. In obese animals, IRI did not aggravate the inflammatory response in obese VAT, probably because of the characteristics of steatotic liver injury and basal state of inflammation in obese adipose tissue.
